# Passive collection of ticks in New Hampshire reveals species-specific patterns of distribution and activity

**DOI:** 10.1093/jme/tjad030

**Published:** 2023-04-08

**Authors:** Natalia Fernández-Ruiz, Agustín Estrada-Peña, Sharon McElroy, Kaitlyn Morse

**Affiliations:** Department of Animal Health, Faculty of Veterinary Medicine, University of Zaragoza, 50013 Zaragoza, Spain; Instituto Agroalimentario de Aragón (IA2), Zaragoza, Spain; Department of Animal Health, Faculty of Veterinary Medicine, University of Zaragoza, 50013 Zaragoza, Spain; Instituto Agroalimentario de Aragón (IA2), Zaragoza, Spain; BeBop Labs, Tick Project, NH 03268, USA; BeBop Labs, Tick Project, NH 03268, USA

**Keywords:** *Ixodes scapularis*, *Dermacentor variabilis*, community science, tick borne pathogens, climate modeling

## Abstract

Ticks and tick-borne diseases are increasing in the United States, including New Hampshire (NH). We report on the findings of an ongoing free crowdsourcing program spanning four years within NH. The date of tick’s submission was recorded along with species, sex, stage, location they were collected (translated into latitude and longitude), the activity the individual was doing when the tick was found, and host species. A total of 14,252 ticks belonging to subclass Acari, family Ixodidae and genera *Ixodes*, *Dermacentor*, *Amblyomma*, and *Haemaphysalis* was recorded from the period 2018–2021 throughout NH. A total of 2,787 *Ixodes scapularis* and 1,041 *Dermacentor variabilis*, were tested for the presence of *Borrelia* sp. (Spirochaetales: Spirochaetaceae), *B. burgdorferi sensu lato*, *B. miyamotoi*, *B. mayonii*, *Babesia microti* (Piroplasmida: Babesiidae), *Anaplasma phagocytophilum* (Rickettsiales: Anaplasmataceae), *Francisella tularensis* (Thiotrichales: Francisellaceae), and *Rickettsia rickettsii* (Rickettsiales: Rickettsiaceae) by PCR. For the *I. scapularis* ticks tested, the pathogen prevalence was 37% *B. burgdorferi s.l*. 1% *B. miyamotoi*, 6% *A. phagocytophilum*, and 5% *Ba. microti*. Only one *D. variabilis* resulted positive to *F. tularensis.* We created state-wide maps informing the differences of ticks as detailed by administrative divisions. *Dermacentor variabilis* peaked in June and *I. scapularis* peaked in May and October. The most reported activity by people with tick encounters was while walking/hiking, and the least was biking. Using the reported distribution of both species of ticks, we modeled their climate suitability in the target territory. In NH, *I. scapularis* and *D. variabilis* have distinct patterns of emergence, abundance, and distribution. Tick prevention is important especially during April–August when both tick species are abundant and active.

## Introduction

Surveillance of disease and vectors of disease is crucial to ensure human and pet safety. Ticks serve as vectors for more than 13 unique human tick-borne illnesses caused by 18 different pathogens in the United States, and there are even more tick-borne pathogens that affect pets and/or livestock (CDC 2015). Throughout the United States, the Centers for Disease Control and Prevention (CDC) receives 30,000 reports of clinical Lyme disease, caused by *Borrelia burgdorferi sensu lato*, but estimations point to 8–12 times underreporting with the actual numbers estimated at 476,000 cases (Kugeler et al. 2021), with the highest numbers concentrated in New England (Schiffman et al. 2018). There is also a concern for other tick-borne diseases that are far less accurately tracked as compared to *B. burgdorferi* (Rochlin and Toledo 2020). To make matters worse, the beginning and end of tick activity/seasons changes according to weather conditions and extends with warmer autumns and winters, making it harder to pinpoint at-risk regions and periods (Wikel 2022). There are numerous records demonstrating that some tick species are spreading to the northern parts of the United States (Eisen and Eisen 2018, Sonenshine 2018, Wikel 2022). Most commonly, climate has been pinpointed as one of the main drivers behind such spread (Ogden et al. 2021), but the presence and abundance of hosts or the landscape features are also important features to account for the spread of ticks (Pfäffle et al. 2013, Talbot et al. 2019).

The distribution of ticks over large territories has been commonly explored by field surveys, which are complex and expensive in nature or conducted at limited temporal or spatial scales. To overcome this hurdle, it has become common to involve community scientists in the surveillance of ticks. Community science, collectively known as the altruistic participation of volunteers in scientific work, is becoming an important ground for several studies dealing with the passive surveillance of the distribution of an organism. This is commonly called ‘public participation in scientific research’, ‘passive surveillance’. or ‘community-based monitoring’. One of the main problems regarding community science is the assessment of the data quality, leading to questioning how good is the data gathered by participants (Cronje et al. 2011, Wiggins et al. 2011, Kosmala et al. 2016, Eisen and Eisen 2021). Issues arose when participants are asked to perform similar tasks as the researchers, producing some data for which they are not prepared (like species identification), even after an introductory course. Community science has been used for passive surveillance of ticks consistently for over one decade and helped to identify the spread of ticks and transmitted pathogens (Ogden et al. 2006, Gasmi et al. 2019, Nelder et al. 2019) or using animals as sentinels (Lee et al. 2019). The validity of passive surveillance has been improved by the multiple applications for mobile devices like smartphones (Hamer et al. 2018, Fernández et al. 2019) that complement the reporting of a parasite with a picture of the specimen and/or the possibility of the mobile device to track its position (an earlier review is available by Madder et al. 2012). To note, in most cases a cell phone does not have enough magnifying power to provide a clear picture of a feeding immature tick, that is commonly identified by a specialist under a stereomicroscope. Thus, the best passive tick surveillance gathers three key pieces of information (Eisen and Eisen 2021, Poh et al. 2022), namely (i) an accurate identification of the tick by specialists, and if necessary, the further processing of the tick(s) for carried pathogen(s); (ii) a basic set of questions prepared by the researcher(s) to gather as much information as possible from the participants; and (iii) coordinates of collection, together with minor details of the record. Although, the influence of socio-economic strata in the altruistic participation, or mistakes in geo-localization remain pertinent drawbacks (Eisen and Eisen 2021).

Several tick species affecting humans have been reported in northeastern United States. Of these, some of the most reported in the region are *Ixodes scapularis* and *Dermacentor variabilis* (Dergousoff et al. 2013, Hahn et al 2016, Sonenshine 2018, Duncan et al. 2021, Wikel 2022). These species increase their spatial range by a variety of reasons, including (but not restricted to) climate trends, changes in land use, and/or (re)colonization by wildlife. Climate trends have been proposed as one of the main traits affecting the distribution of the ticks. Efforts are addressed to capture their environmental niche and project the results into the territory (Alkishe et al. 2021). Spatial modeling can thus predict the probability of the presence of a tick (Laniak et al. 2013). The environmental niche is defined by using records of tick coordinates linked with variables like temperature and the amount of water in the ground or the air and matching the known distribution of the species with the preferred range of the environmental variables (Estrada-Peña et al. 2013).

New Hampshire (NH) is a northeastern state of United States and one of the places with highest incidence of Lyme disease, Anaplasmosis, and Babesiosis in USA, as reported by the CDC (2021). This study aims to analyze the ticks submitted to BeBop Labs (https://www.beboplabs.org), a non-profit organization that is filling in the gaps of ticks and tick-borne disease surveillance within NH. We summarize the ticks collected from humans, pets, and other domestic animals, pinpointing conclusions about the distribution and activity period(s) of the ticks, the most common situations of risk, the pathogens carried as detected by PCR, and a preliminary modeling of the two most reported species, namely *I. scapularis* and *D. variabilis*.

## Methods

### Tick Submission and Identification

Ticks started to be collected by residents in NH, in June 2017 through a free program. The program was advertised through partnerships with Plymouth State University, the University of NH, NH camps, and similar organizations, as well as through local newspaper articles and social media. Submitted ticks were received by postal mail along with a questionnaire. Since the program is still ongoing, this study reports the data between the years 2018 and 2021. In 2017, there were fewer ticks submitted, and the accompanying questionnaire was different. Due to the SARS-CoV-2 pandemic in 2020, the participation could have also been affected, but the program was still running. Ticks were received live, dead, taped to paper, or in alcohol, but immediately upon receipt ticks were kept dehumidified and at room temperature until identification and pathogen testing, a method that has been shown to preserve DNA (Bonnet et al. 2010). For each tick, we recorded the date of collection, and we identified species, sex, stage, location in which the ticks were collected (specific as town and street address, and/or latitude and longitude), the activity that the host was doing when the tick was found (for human hosts), if the tick was crawling or biting, and the species of host (i.e., human, dog, etc.). Although each submission was annotated with the exact date of finding and/or removal, weekly intervals (together with a mention to the month and the season) provided a better overview of the period of tick activity. Tick species identification was based on published identification keys (Clifford et al. 1961, Brinton et al. 1965, USDA 1976, Keirans et al. 1978, 1998a, Yunker et al. 1986, Durden et al. 1996). Within the results, we have also included 111 ticks obtained by shared information from Ticknology, Fort Collins, CO, for the year 2019, and the publicly available tick submission information from MedZu, Inc., Tick Report, Amherst, MA (formerly within University of Massachusetts Amherst Laboratory of Medical Zoology) for the complete period (years 2018–2021) available at: https://www.tickreport.com/stats. Thus, some species of ticks were also identified using PCR targeting specific genes from individual tick species and performed by these laboratories. We focused our analyses on *I. scapularis* and *D. variabilis*, the most prominent tick species received, yet we also identified a few other tick species (see below, Results, Table 1) and they were not analyzed further.

**Table 1.** The total number of submitted ticks by year in the period 2018–2021 by volunteer participants in New Hampshire

Year	*Ixodes scapularis*	*Dermacentor variabilis*	*Ixodes cookei*	*Ixodes marxi*	*Ixodes pacificus*	*Ixodes* spp.	*Amblyomma americanum*	*Dermacentor albipictus*	*Dermacentor* spp.	*Haemaphysalis longicornis*	Unidentified	Total
2018	516	1,090	4	2	0	0	2	0	0	0	0	1,614
2019	1,314	4,407	5	2	1	0	3	2	1	0	14	5,749
2020	1,141	878	5	0	0	1	1	1	0	0	14	2,041
2021	1,286	3,501	3	0	0	0	1	2	9	1	45	4,848
Total	4,257	9,876	17	4	1	1	7	5	10	1	73	14,252

#### Location of tick.

Participants recorded location to the best of our knowledge to where the tick was found. Explanations of the activity done just before noticing the bite of the tick, including details like ‘biking between A and B (several kilometers)’ could not provide reliable coordinates of the exact place of bite. Therefore, we included the coordinates as submitted. All the results regarding the number of ticks submitted and identified were mapped at the level of the administrative perimeter of each town in NH, as provided by GRANIT, the statewide repository of geographical information (https://www.granit.unh.edu, last accessed Dec 2021). We adopted this mapping procedure because the ZIP codes are too broad, sometimes overlapping, and not contiguous to accurately represent the spatial variability of the collected ticks.

In viewing the use of the natural vegetation areas over the State, as available in the US Environmental Protection Agency (http://www.epa.gov/wed/pages/ecoregions.htm, last accessed Dec 2021), we determined these regions are divided into three hierarchical levels. Level I is the coarsest level, dividing North America into 15 ecological regions. Level II divides the continent into 50 regions. At Level III, there are 181 ecological regions for North America. We used the level III, just to realize that there are only 3 of these ecological regions over New Hampshire, therefore the vegetation areas were too large to provide a meaningful interpretation of the results.

We used the index Moran’s *I* to estimate two different values of spatial autocorrelation, namely (i) the number of ticks received from each administrative division in NH, and (ii) the number of residents in each division. Low values of Moran’s *I* (near −1) typically mean randomness of data or no spatial association, high values (near +1) are indicative of clustering thus consistent with a spatial association. A high clustering of the number of specimens received according to administrative divisions may be indicative of (i) there is a patchy suitable habitat or (ii) the participants submitting ticks are highly clustered in the space: We also carried out a linear correlation to check if there is a relationship between the number of people living in each administrative division and the number of ticks submitted, which would be indicative of the simple rule ‘more ticks from a site because more residents in such site’. Moran’s *I* was calculated in R (R Core Team 2022) using the code provided by Brundson and Comber (2022) for the book ‘An Introduction to Spatial Analysis in R’; code is available on Internet (https://bookdown.org/lexcomber/brunsdoncomber2e/) and is not part of the original book.

#### Tick hosts.

Due to the wide variety of hosts reported by the correspondents, we separated them by humans, pets (dogs and cats), farm animals (cattle, horses, sheep, goat), deer, other, and blank (no response given). Other refers to inanimate objects such as couch, counter, wall, or floor. We disregarded the status of the tick (i.e., feeding or crawling on the host) and considered that each one represents a record. Most likely, the discovery of a crawling tick was because of an increased awareness of the person; thus, preventing the bite.

Total numbers of ticks are presented in all tables, percent of total is calculated by dividing the individual number by total multiplied by 100. Statistical tests are elaborated further in the results section and performed using Microsoft Excel, typically a one-tailed homoscedastic T-test to assess differences in average tick numbers.

### Detection of Pathogens

All life stages of *I*. *scapularis* and *D. variabilis* were tested for the presence of the most common tick-borne pathogens reported in New England (i.e., Rounsville et al. 2021). Ticks were tested by fee for service laboratories, following each laboratories’ protocols (Dykstra et al. 2020). The ticks collected in 2018–2020 were tested by both Ticknology, Fort Collins, CO and MedZu, Inc., Tick Report, Amherst, MA (formerly within University of Massachusetts Amherst Laboratory of Medical Zoology) and ticks collected in 2021 were tested by MedZu. Ticknology adhered to the following protocol: ticks were transferred to 1.5 ml microcentrifuge tubes before resuspension in Buffer ATL (Qiagen) and homogenized manually according to the manufacturer’s instructions. *Borrelia* species*, B. burgdorferi s. l., B. miyamotoi, B. mayonii, Babesia microti,* and *Anaplasma phagocytophilum* were detected in two multiplex TaqMan PCR assays targeting different genes. *Dermacentor variabilis* was also tested for *Francisella tularensis* and *Rickettsia rickettsii.* One multiplex also targeted *I. scapularis* actin, which acts as a positive control for the DNA purification and PCR reaction. Both positive and negative controls for each PCR target were also performed with each 96 well plates representing ~3% of the total tests performed. MedZu protocol is published in numerous reports (Xu et al. 2016, 2019, 2021, Dykstra et al. 2020, Milholland et al. 2021) and is as follows. Briefly, DNA was extracted from each tick using the Epicenter Master Complete DNA and RNA Purification Kits (Epicenter Technologies, Madison, WI) following the manufacturer’s protocols. The selected pathogens were detected by a multiplex TaqMan PCR assay targeting different genes (Xu et al. 2016, 2019) in 20 μl reaction volumes using Brilliant II qPCR Master Mix (Agilent, La Jolla, CA). Xu et al. 2016 describe the cycling conditions 10 min at 95°C with 40 15 s cycles at 95°C and 1 min at 60°C as well as the primers for *B. microti* tubulin (Forward GATTTGGAACCTGGCACCATG, Reverse AATGACCCTTAGCCCAATTATTTCC) and *A. phagocytophilum* MSP2 (Forward ATGGAAGGTAGTGTTGGTTATGGTATT, Reverse TTGGTCTTGAAGCGCTCGTA). Xu et al. (2019) reiterated those primers and further provided sequences to differentiate *I. scapularis* (Forward TGCGTTTTCTTTGAGCAAATGCACGAG, Reverse GTACGGGATTTTCCACAAACGGTATCCA) from *I. pacificus* (Forward CTCGGAGCAAGTACGGAGGTAG, Reverse TTTCCACAAAACGGTCGCCATC) and the detection of *B. burgdorferi s.l.* ospA (Forward ATAGGTCTAATATTAGCCTTAATAGCAT, Reverse AGATCGTACTTGCCGTCTT) and *B. miyamotoi* glpQ (Forward GACATAGTTCTAACAAAGGACAATATTCC, Reverse TCCGTTTTCTCTAGCTCGATTGG). The protocols of submission followed in this study precluded the preparation of samples of ticks of the same species and sex to prepare pools and calculate the Minimum Infection Rate (MIR) as already done in other studies (Burket et al. 1998, Wójcik-Fatla et al. 2011).

### Ticks According to Human Activities

We received a variety of responses regarding the activity that the host was doing when the tick was found because we allowed for an open-text question. To compile responses, we first disregarded any responses to the activity section on any host other than humans. Then we categorized responses together by (i) identifying and defining the categories as they appear in the questionnaires, (ii) generating a list that recompiles all the activities written by the participants into a smaller set of categories, and (iii) we totaled all years together to achieve higher numbers and analyzed by the percent of total number of tick species versus human activity category and in a heat map with number of ticks per human activity category during weeks of the year. We summarized the categories below:

1. **Field and Wilderness:** Deep contact with fields beyond a backyard and not on trails, including camping and anything with the world ‘field’ in it. We consider these activities the deepest contact of humans with the wild areas. Examples: archery, birding, bug catching, bushwhack hiking, camping, campfire, field games, geocaching, haying, hunting, orienteering, picking wild blueberries, surveying, or walking through woods or long grass.
2. **Walking/Hiking:** Walking along designated trails within a rural setting, not urbanized (see category 5) or with pets (see category 7). Examples: hiking on a trail, nature walk, rock climbing, walking on trails, trail work, walking across backyard or woods, trail running.
3. **Backyard activities:** Defined as activities done within a backyard of a home or a workplace. Examples: chilling in backyard, clearing brush, fence repair, gardening, home repairs, landscaping, masonry, mowing lawn, at a park, planting or harvesting garden, playing in yard, raking leaves, recess, splitting wood, weeding, yardwork.
4. **Biking**: Moving fast along a trail on an exposed vehicle. Examples: biking, four-wheeling.
5. **Urbanized**: Activities within an urbanized setting, including within shared public spaces. Examples: 9-square, baseball, basketball, getting mail, loading car, picking up trash along road, playground, ropes course, tennis, unloading groceries, walking along road.
6. **Indoor:** The participants indicated that the tick was noticed indoors most likely after any kind of other unreported or unnoticed previous activities. Defined as found inside a building, including anything eating related such as having breakfast, lunch, or dinner. Examples: art, bathroom, in bed, in cabin, changing clothes, classes, cleaning, dinner, driving, eating, in house, laundry, office work, playing music, reading, resting, school, showering, sitting, sleeping, tick checks, and watching TV.
7. **Animal related**: Defined as activities with animals including pets. Examples: barn chores, beekeeping, dog walk, farming, feeding pig, hiking with dogs, raking goat pen, and tending chickens.
8. **Water related**: Activities around water. Examples: boating, fishing, gold panning, kayaking, sitting on beach, and swimming.
9. **Eliminated:** Responses such as AP, arm, back, belly, biting, ear, hair, hand, neck, NP, on clothes, S, shoulder, under pants, and wrist were not considered an activity.
10. **Blank**. No activity recorded.

### Modeling

For modeling the potential distribution of *I. scapularis* and *D. variabilis* in NH we used the coordinates provided by the participants when submitting the ticks. As explanatory variables, we used the first three coefficients of a harmonic regression of the mean annual temperature, soil humidity, and atmospheric water vapor deficit between the years 1980 and 2018. Climate data were obtained from the TerraClimate public repository (http://www.climatologylab.org/terraclimate.html, last accessed Mar 2021). The procedure of the harmonic regression was proposed by Estrada-Peña et al. (2014) as a method to decompose the series of monthly climate values, improving time resolution and retaining the ecological meaning. These authors also demonstrated that the coefficients of the regression are the best explanatory variables (i.e., the model predictors) of the environmental suitability of a territory for a tick, because they represent average annual values, their seasonality, and the amplitude for each season. Many coefficients could be calculated, but in practical terms, the first three provide an adequate reliability for outlining the climate suitability. Complete details and examples can be found in the original publication (Estrada-Peña et al. 2014).

Together with the series of monthly environmental variables, including mean temperature, soil humidity, and water vapor deficit between 1980 and 2018, the coordinates for both *I. scapularis* and *D. variabilis* were used to train the model. These coordinates may not always correspond with the actual site of collection, and some of them may represent the coordinates of a persons’ home. In any case, given the area of New Hampshire and the high number of ticks received, we decided to ignore the negligible bias produced by these probably incorrectly allocated records. Anyway, we should expect more tick samples submitted from areas where either the tick is abundant and/or where the human population is high. We addressed this issue by (i) removing the repeated coordinates in modeling (i.e., many submissions from the same pair of coordinates) and (ii) calculating if there is a significant higher number of ticks received from areas with larger population. We did a simple linear regression (and calculated its significance, *P*) using Microsoft Excel between the population of each administrative division in NH against the number of ticks submitted from that division, assuming that the effort for tick detection and submission was the same in every territory.

We independently modeled the presence of each tick species using the niche modeling algorithm MaxEnt integrated in the 'dismo’ package (Hijmans et al. 2020) for R (R Core Team 2022). Models were developed with linear and quadratic features, using 10,000 background points (in which the ticks have not been reported). Each model was replicated 100 times per species, using 70% of points for training purposes, removing duplicate coordinates, and selecting the best model regarding the value of the Boyce’s index reported to perform better that the commonly used Area Under the Curve AUC (Hijmans et al. 2020). Cross-validation was used to compare the resulting models, partitioning the data into replicate folds, with each fold used in turn to test the model. The regularization multiplier was set to 1. Finally, the results from the models were plotted following the administrative divisions of New Hampshire, to follow actual tick records as mentioned before.

## Results

### Tick Species Identified (Years 2018–2021)

We received a total of 14,252 ticks in the period 2018–2021, collected in 259 different sites throughout NH (Table 1). The most submitted species of ticks were *I. scapularis* at 4,257 and *D. variabilis* at 9,876 specimens, together totaling 14,133. *Dermacentor variabilis* was approximately twice as abundant as *I. scapularis* and statistically different at *P*-value 0.08 for one-tailed homoscedastic T-test. A few specimens of *Amblyomma americanum* (7), *I. cookei* (17), *I. marxi* (4), *I. pacificus* (1) D. *albipictus* (5), and one *Haemaphysalis longicornis*, as well as 10 undetermined *Dermacentor* spp. were also submitted. A total of 73 ticks were damaged enough to prevent its identification. The life stages are indicated only for *I. scapularis* and *D. variabilis* in Table 2.

**Table 2.** The hosts from which *I. scapularis* and *D. variabilis* received were found, arranged by years, and separated as adults, nymphs, or unknown stages

Host	2018						2019						2020						2021						Total #
	I. scapularis			D. variabilis			I. scapularis			D. variabilis			I. scapularis			D. variabilis			I. scapularis			D. variabilis			
	Nymph	Adult	Unk. h	Nymph	Adult	Unk. h	Nymph	Adult	Unk. h	Nymph	Adult	Unk. h	Nymph	Adult	Unk. h	Nymph	Adult	Unk. h	Nymph	Adult	Unk. h	Nymph	Adult	Unk. h	
Humans^a^	7	269	1	0	24	578	25	149	0	1	2,702	28	7	221	0	0	572	2	19	139 (54 with dog)^g^	1	0	1705 (19 with dog)^g^	78	**6,601**
Pets^b^	0	25	0	0	30	61	1	188	2	0	682	4	3	219	0	0	106	0	3	530	1	0	842	70	**2,767**
Farm animals^c^	0	0	0	0	0	0	0	4	0	0	55	0	0	0	0	0	4	0	0	25	0	0	38	0	**1,26**
Deer^d^	0	0	0	0	0	0	0	0	0	0	0	0	0	0	0	0	0	0	0	62	0	0	0	0	**62**
Other^e^	0	0	1	0	0	13	0	2	0	0	72	0	0	2	0	0	41	0	0	2	0	0	197	0	**330**
Blank^f^	0	0	213	0	3	381	191 (5 larva)	691	56	1	803	59	47 (7 larva)	621	14	1 larva	148	4	79 (4 larva)	365	2	1	546	5	**4,247**
Total	**7**	**294**	**215**	**0**	**57**	**1,033**	**222**	**1,034**	**58**	**2**	**4,314**	**91**	**64**	**1,063**	**14**	**1**	**871**	**6**	**105**	**1177**	**4**	**1**	**3,347**	**153**	**14,133**
Grand totals	516			1,090			1,314			4,407			1,141			878			1,286			3,501			

^a^Total 892 *I. scapularis* and 5,709 *D. variabilis* found on humans for all years.

^b^Total 972 *I. scapularis* and 1795 *D. variabilis* found on pets for all years.

^c^Total 29 *I. scapularis* and 97 *D. variabilis* found on farm animals for all years.

^d^Total 62 *I. scapularis* and 0 *D. variabilis* found on deer for all years.

^e^Other indicates inanimate objects such as couch, counter, wall, and floor. Total 7 *I. scapularis* and 323 *D. variabilis* found on other for all years.

^f^Blank indicates a host was not given. Total 2,295 *I. scapularis* and 1,952 *D. variabilis* for all years.

^g^Host response indicated ticks were found on both dog and human.

^h^Unk: Unknown, referring to an unrecorded life stage.

We received a total of 3,568 adults (~84%), 382 nymphs (~9%), 16 larvae (~less than 1%), and 291 unrecorded life stages (~7%) *I. scapularis* (Table 2). We received a total of 8,589 adults (~87%), 3 nymphs (~less than 1%), 1 larva (~less than 1%), and 1,283 unrecorded life stage (~13%) *D. variabilis* (Table 2). For all years, most ticks were collected on humans, representing 892 *I. scapularis* and 5,709 *D. variabilis* totaling 6,601 ticks. *Dermacentor variabilis* were found on humans 5 times more than *I. scapularis* (*T* = 4,844.22; *P* = 0.06 for one-tailed homoscedastic T test). We also received 4,247 ticks without a recorded host, including 2,295 *I. scapularis*, (including most of the nymphal *I. scapularis*) and 1,952 *D. variabilis* (*T* = 1,108.19; *P* = 0.46). Up to 972 *I. scapularis* and 1,795 *D. variabilis* were found on pets (*T* = 2,219.54; *P* = 0.24), 29 *I. scapularis* or 97 *D. variabilis* on farm animals (*T* = 2,988.01; *P* = 0.17); most of these were adult ticks. Only 7 *I. scapularis* were found on other hosts, as compared to 323 *D. variabilis* (*T* = 4,777.75; *P* = 0.065) indicating the unique questing habits of *D. variabilis* are on inanimate objects. Only adult *I. scapularis* were found on deer. Looking at the numbers of ticks from both Table 1 and 2, a human host is more likely to encounter a *D. variabilis* ticks than *I. scapularis* (*T* = 5,6779.44; *P* = 0.06) (including finding them in a general human environment, the ‘other hosts’), but having a pet (*T*: 5,118.29; *P* = 0.24) results in about an equal chance of encounter for both species of ticks.

### Tick Distribution

**Fig. 1.**
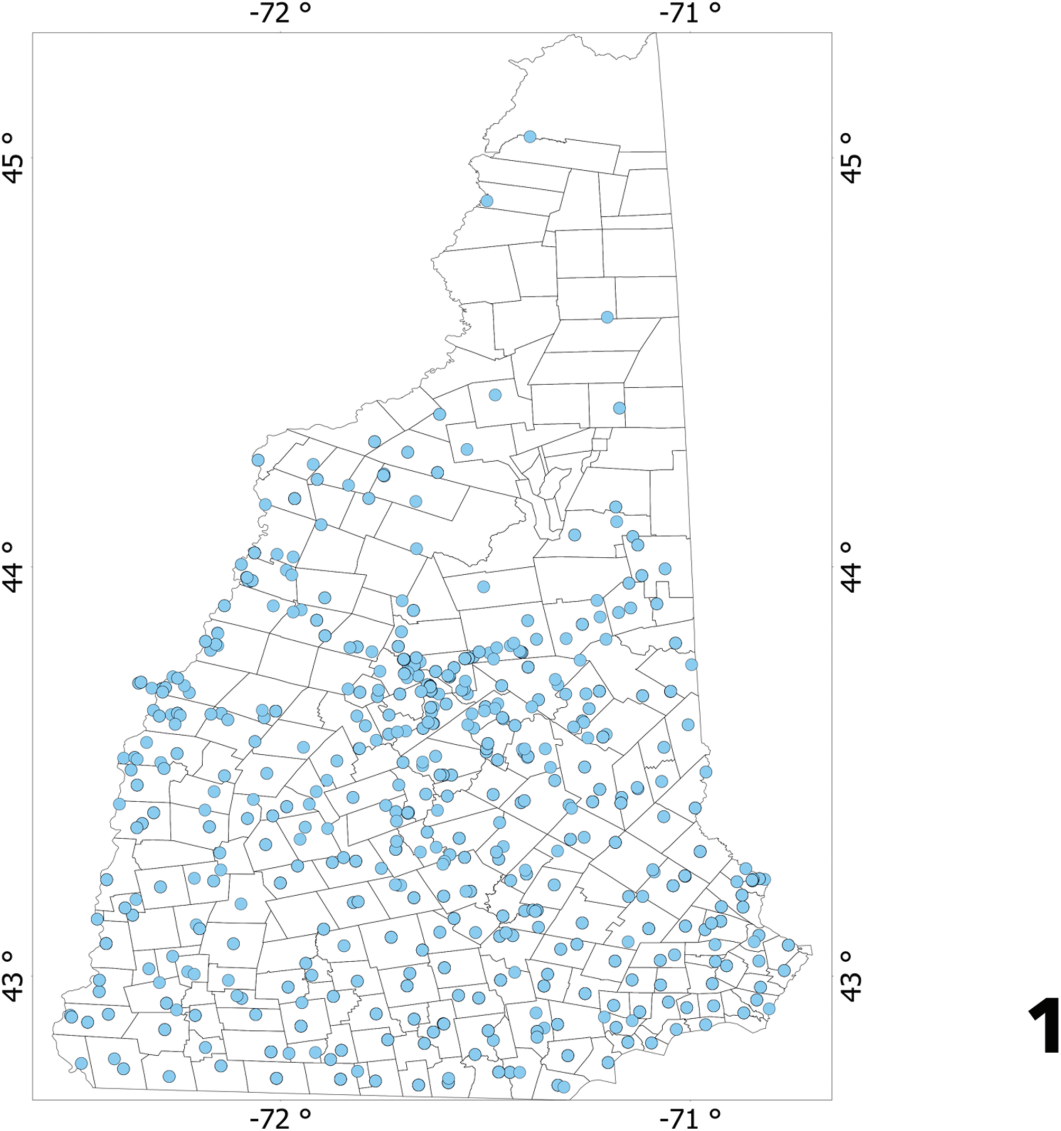
The spatial distribution of the sites in New Hampshire from which ticks were submitted for this study, according to the coordinates provided by the participants. The dots only indicate occurrence but not abundance. In some cases, specimens were submitted without coordinates but with the name of a town. In these cases, coordinates of the town were obtained and plotted. The map shows where participants have concentrated as well as the sites in the northern parts of the State from where no ticks were submitted.

Ticks were collected primarily in the southern parts of the state (Fig. 1) showing a clear contrast with the probable absence of both *I. scapularis* and *D. variabilis* in the northern region of the state. We also noticed there is also a small cluster of submissions in the upper middle of the state where the project started.

**Fig. 2.**
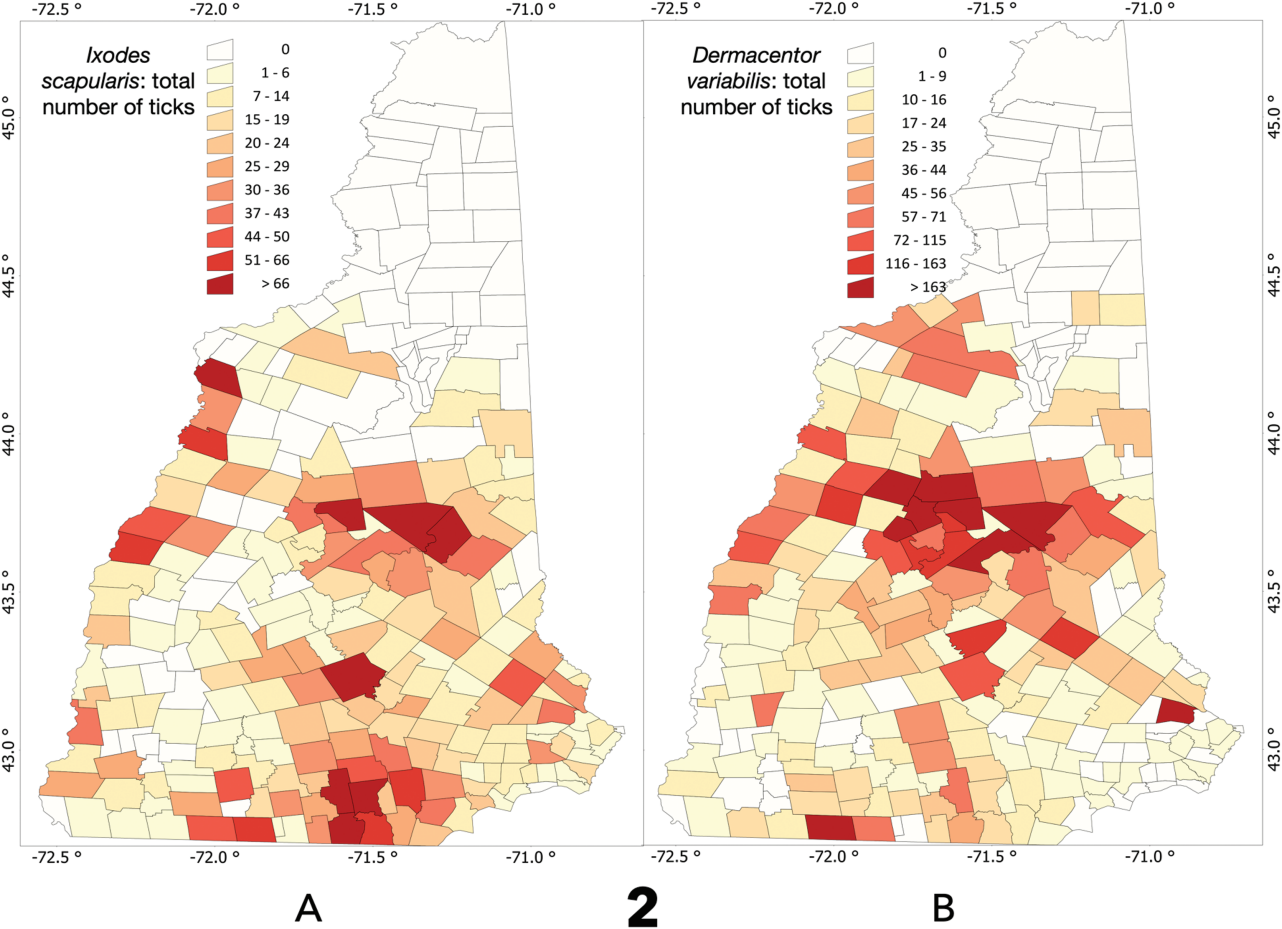
The distribution of ticks mapped in New Hampshire according to the number of submitted specimens in the period 2018–2021 at the level of ‘city boundaries’. The distribution of *I. scapularis* (Fig. 2A), with a total number of 3,642. The distribution of *D. variabilis* (Fig. 2B), with a total number of 6,494. The legend reflect the total number of specimens received from each administrative division.

The number of *I. scapularis* and *D. variabilis* were aggregated at the spatial level of administrative divisions (Fig. 2). The total number of mapped *I. scapularis* is 3,642; the value for total *D. variabilis* is 6,494. The values in the map reflect the sum of ticks received from each administrative division. This provides a better pattern for capturing the distribution and abundance of the ticks than the pure dot or the aggregation of data at the level of county.

Other than the general rule of their absence in the northern regions, both *I. scapularis and D. variabilis* are noticed at variable numbers and unique clusters. *Ixodes scapularis* seems to prefer areas near the south and coastal parts of the state, but also reach high abundance in sites of central parts of the state, as well as being more abundant on the western border of NH. Although *D. variabilis* is found more northern than *I. scapularis* it is also absent for the most northern regions of the state. Some *D. variabilis* were submitted from the southern and southeastern NH, yet the highest number of specimens were received from the central parts of the state into the western border. The central cluster of *D. variabilis* is more prominent than for *I. scapularis*. Values of Moran’s I (*I. scapularis*: −0.204, *P* < 0.01; *D. variabilis*: −0.228, *P* = 0.09) confirmed that both species have a random distribution with no spatial association. Values of Moran’s I for the population of administrative were also clearly negative (−0.399, *P* = 0.07). The correlation between the population of each administrative division of NH and the total number of ticks (including both species) submitted is low (*R*^2^: 0.401, *P* = 0.22). Thus, ticks were received from sites where they are more abundant, or awareness is higher, not from sites where a larger human population (and thus more participants) resides.

### Seasonal Activity of Ticks

**Fig. 3.**
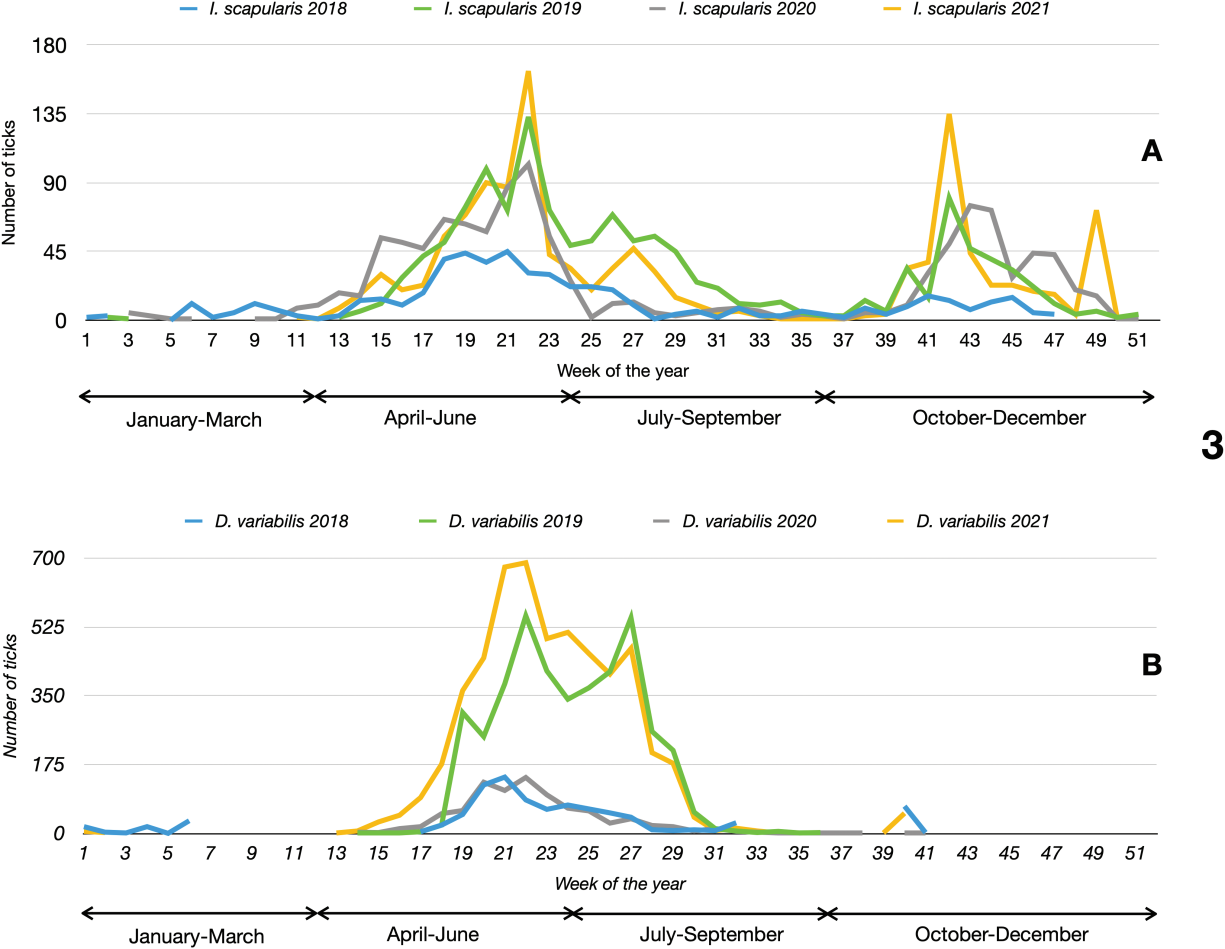
The seasonal pattern of abundance of *I. scapularis* (Fig. 3A) and *D. variabilis* (Fig. 3B) in New Hampshire at a time resolution of one week, with indications of the month of the year, based on the number of each specimen submitted by the participants. Different lines indicate the different years 2018–2021.

The weekly activity of submitted *I. scapularis* or *D. variabilis* is presented in Fig. 3. It was not possible to separate stages and activity because most samples were adults (probably because the larger and noticeable size); we considered that extrapolations of lack of immatures’ in this context could be statistically risky. Activity patterns, considering all stages together, were consistent among years, but abundance varied for each species and for each year. The highest abundance of *I. scapularis* concentrated in weeks 13–31, or April–June and autumn to winter, from weeks 40–49, or September–November. Looking closely at the differences from 2018 to 2021, the time that increasing numbers of *I. scapularis* were collected progressively gets earlier each year. In 2018, *I. scapularis* submissions began at week 16–17, 2019 at week 15, and 2020 and 2021 at week 13. While *I. scapularis* activity was bimodal, the seasonal activity of *D. variabilis* had only one major peak corresponding to approximately week 21 with a total period of submitted ticks from week 16 to 31 or April through August. Submissions varied by year, but every other year more *D. variabilis* were submitted. Looking at these four years of data, it appears *D. variabilis* comes out in much larger numbers every other year. The numbers of *D. variabilis* collected in 2018 and 2020 were just less than 1,000 and in 2019 and 2021 were greater than 3,000. In any case, weeks 16–31 or April–August should be considered of high risk for humans in NH to encounter both *I. scapularis* and *D. variabilis* ticks since this time of the year concentrates the maximum activity of both most prominent tick species.

**Fig. 4.**
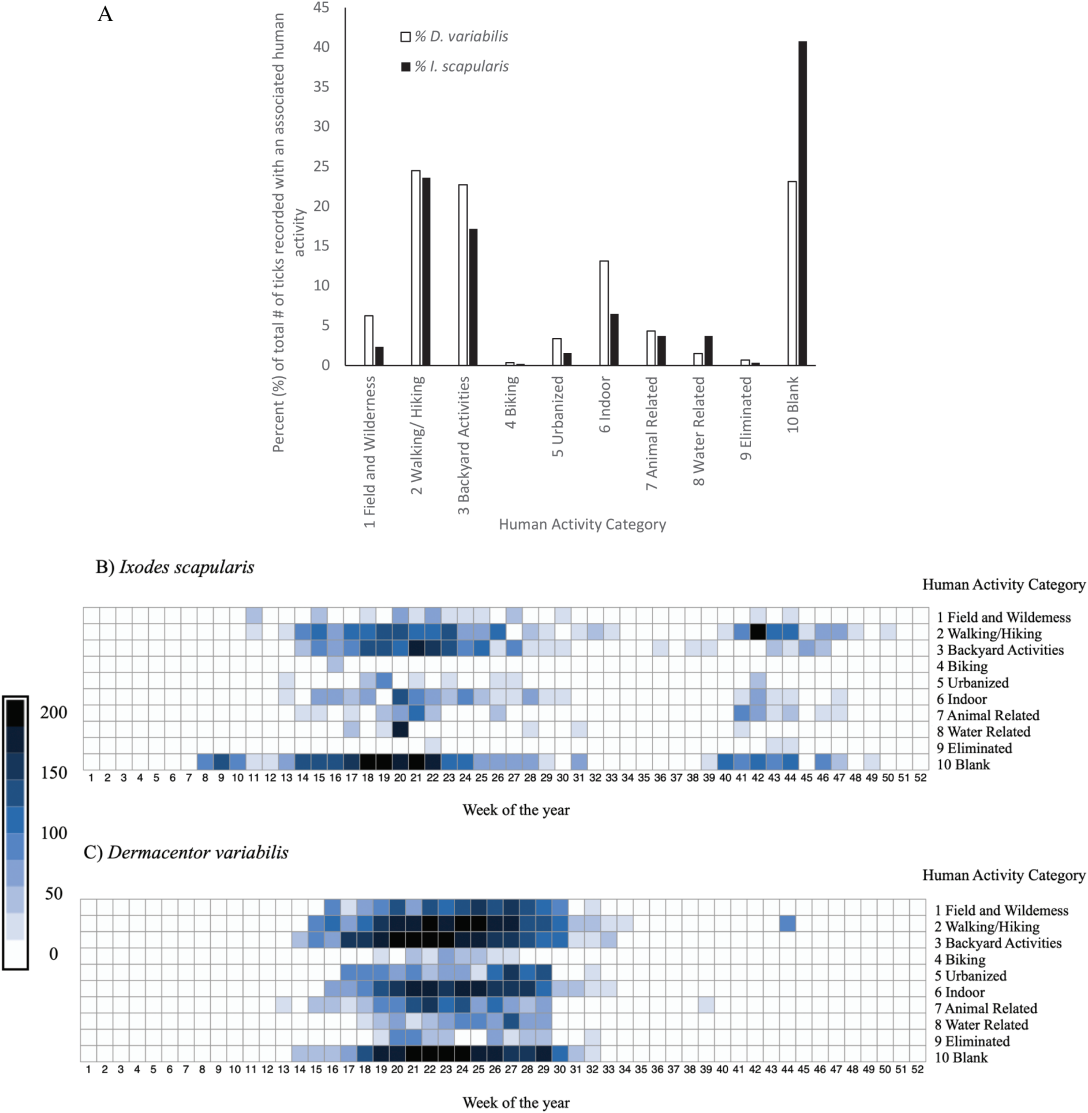
Totaled ticks found per human activity over years 2018–2021. Figure 4A depicts the percent of *I. scapularis* (black) and *D. variabilis (white)* found for each associated category of human activity. Figure 4B and C depicts heat maps for the number of *I. scapularis* (Fig. 4B) and *D. variabilis* (Fig. 4C) shown according to the week of the year and the associated category of human activity. Scale is from 0 to 200 darker shades representing more numbers of ticks.

Human activities associated with the finding of *I. scapularis* (890 records) or *D. variabilis* (5,709 records) ticks (a total of 6,599 records) are depicted in Fig. 4. Figure 4A depicts the percentage of the total ticks recorded associated with a human activity category for each tick species for each human activity category. Figure 4B and C maps the number of ticks, *I. scapularis* in Fig. 4B and D*. variabilis* in Fig. 4C along a heat map with the associated human activity category by week of the year. There was a significant portion of ticks submitted to our project that were not associated with a human activity (category 10), corresponding to 23% *D. variabilis* and most of the *I. scapularis* or 41% (Fig. 4A). Despite this observation, the group ‘walking/hiking’ (category 2) is associated with the highest tick encounters. Respectively, 24% of *I. scapularis* (Fig. 4A) occurring throughout weeks 16–25 and even higher tick numbers in the weeks 39–48 (fall season) were found while ‘walking/hiking’ (category 2, Fig. 4B). Twenty-five percent of *D. variabilis* were collected while ‘walking/hiking’ (category 2, Fig. 4A), and the peak was observed within weeks 16–31 (Fig. 4C). The next highest-risk activity is related to ‘backyard activities’ (category 3) for which we found 23% likelihood of finding *D. variabilis* from weeks 17–30 and 17% *I. scapularis* from weeks 14–25. It is twice as likely to find *D. variabilis* than *I. scapularis* while performing activities in fields and wilderness (category 1, 6%–2%, respectively), urbanized (category 5, 3%–1.6%, respectively), or indoors (category 6, 13%–6.5%, respectively). In activities around water (category 8) people were twice as likely to find *I. scapularis* (3.7%) than *D. variabilis* (1.5%). It is necessary to note that biking (category 4) is the category with the lowest encounter for finding both *D. variabilis* and *I. scapularis*. A low percentage of ticks, mostly *D. variabilis* were not able to be categorized by participants.

### Pathogens Detected in the Submitted Ticks

The search for pathogens was focused on the most common tick-borne pathogens reported in New England. We tested 2,787 *I. scapularis* and 1,041 *D. variabilis*. About 1,191 *I. scapularis* were positive for a pathogen (~43% *I. scapularis* carried a pathogen) and only 1 *D. variabilis* was positive for *F. tularensis* in 2019 (~0.1% *D. variabilis* carried a pathogen). Results for the pathogens harbored by *I. scapularis* are included in Table 3. In total for all years and including nymphal and adult stages, *Borrelia* spp. was found in 38% of the tested *I. scapularis* (Table 3). To note, *B. mayonii* and *R. rickettsii* were not found in any tick. The complex of species *B. burgdorferi s.l*., has been recorded in 37% of the processed *I. scapularis*. The recurrent–fever agent, *B. miyamotoi* has been found in a scarce 1% of processed ticks (37 specimens). On the other hand, both *A. phagocytophilum* and *Ba. microti* had low prevalence values, around 5%–6% of the *I. scapularis* processed (169 and 148, respectively). We also found that out of all *I. scapularis* collected through 2018–2021, 7% (204 ticks) were co-infected by any pair, triple, or quadruple combination of the pathogens mentioned. We also included in Table 3 the breakout of pathogen detection among adult and nymph *I. scapularis* ticks per season of each year 2018–2021. In the combined totals, including all years, adult ticks carried on average for all years 42% *Borrelia* spp., 41% *B. burgdorferi s.l*., 1% *B. miyamotoi*, 7% *A. phagocytophilum*, and 6% *Ba. microti*. Nymphs carry on average 18% *Borrelia* spp., 17% *B. burgdorferi s.l*., 1% *B. miyamotoi*, 4% *A. phagocytophilum*, and 3% *Ba. microti*.

**Table 3.** Prevalence of the five pathogens found in adult and nymph *I. scapularis* ticks in New Hampshire by season

Agent^a^		Number of positive ticks (% positive)												
		2018			2019			2020			2021			Grand totals (% positive)
		Spring^b^	Fall^b^	Total^c^	Spring^b^	Fall^b^	Total^c^	Spring^b^	Fall^b^	Total^c^	Spring^b^	Fall^b^	Total^c^	
**Total Tested # of ticks**	**Total**	**390**	**121**	**512**	**616**	**284**	**901**	**486**	**287**	**773**	**232**	**367**	**601**	**2,787**
	**Adult**	**281**	**10**	**292**	**459**	**214**	**674**	**455**	**260**	**715**	**158**	**341**	**501**	
	**Nymph**	**5**	**2**	**7**	**124**	**41**	**165**	**31**	**20**	**50**	**73**	**21**	**94**	
*Borrelia*	Total	159 (41)	35 (29)	**195 (38)**	217 (35)	111 (39)	**328 (36)**	182 (37)	131 (46)	**313 (40)**	84 (36)	149 (41)	**235 (39)**	**1071 (38)**
	Adult	118 (42)	4 (40)	**123 (42)**	189 (41)	95 (44)	**284 (42)**	175 (38)	126 (48)	301 (42)	73 (46)	142 (42)	217 (43)	
	Nymph	3 (60)	0 (0)	**3 (43)**	17 (14)	8 (20)	**25 (15)**	7 (23)	5 (25)	12 (24)	10 (14)	6 (29)	16 (17)	
*Borrelia burgdorferi sensu lato*	Total	157 (40)	32 (26)	**190 (37)**	214 (37)	109 (38)	**323 (36)**	177 (36)	129 (45)	**306 (40)**	81 (40)	139 (35)	**222 (37)**	**1041 (37)**
	Adult	116 (41)	3 (30)	**120 (41)**	187 (41)	93 (43)	**280 (42)**	170 (37)	124 (48)	**294 (41)**	72 (46)	134 (39)	**208 (42)**	
	Nymph	3 (60)	0 (0)	**3 (43)**	16 (43)	8 (20)	**24 (15)**	7 (23)	5 (24)	**12 (24)**	9 (12)	5 (24)	**14 (15)**	
*Borrelia miyamotoi*	Total	6 (2)	3 (2)	**9 (2)**	6 (1)	2 (1)	**8 (1)**	7 (1)	5 (2)	**12 (2)**	2 (1)	6 (2)	**8 (1)**	**37 (1)**
	Adult	6 (2)	3 (30)	**6 (2)**	5 (1)	2 (1)	**7 (1)**	7 (2)	5 (2)	**12 (2)**	1 (1)	4 (1)	**5 (1)**	
	Nymph	0 (0)	0 (0)	**0 (0)**	1 (1)	0 (0)	**1 (1)**	0 (0)	0 (0)	**0 (0)**	1 (1)	1 (5)	**2 (2)**	
*Anaplasma phagocytophilum*	Total	26 (7)	6 (5)	**32 (6)**	21 (3)	18 (6)	**37 (4)**	26 (5)	16 (6)	**43 (6)**	22 (9)	35 (10)	**57 (9)**	**169 (6)**
	Adult	23 (8)	0 (0)	**23 (8)**	15 (3)	16 (7)	**31 (5)**	23 (5)	16 (6)	**39 (5)**	19 (12)	34 (10)	**53 (11)**	
	Nymph	0 (0)	0 (0)	**0 (0)**	4 (3)	2 (5)	**6 (4)**	3 (10)	1 (5)	**4 (8)**	2 (3)	0 (0)	**2 (2)**	
*Babesia microti*	Total	30 (8)	5 (4)	**35 (7)**	25 (4)	11 (4)	**36 (4)**	18 (4)	24 (8)	**42 (5)**	12 (5)	23 (6)	**35 (6)**	**148 (5)**
	Adult	24 (9)	1 (10)	**25 (9)**	20 (4)	3 (4)	**29 (4)**	18 (4)	24 (9)	**42 (6)**	10 (6)	21 (6)	**31 (6)**	
	Nymph	0 (0)	0 (0)	**0 (0)**	4 (3)	1 (3)	**5 (3)**	0 (0)	0 (0)	**0 (0)**	2 (3)	2 (10)	**4 (4)**	
Coinfected^d^	Total	37 (9)	7 (6)	**44 (9)**	30 (5)	14 (5)	**44 (5)**	23 (5)	29 (10)	**52 (7)**	24 (10)	40 (11)	**64 (11)**	**204 (7)**

^a^ *B. mayonii* was not detected in any tick.

^b^Spring includes January through July and Fall includes August through December. The grand totals for Spring and Fall include all stages i.e., larvae and unknown are included.

^c^Grand totals for each year include stage as indicated or if total all stages including larvae and unknown, as well as all months including blanks.

^d^Coinfection is defined as any tick that contains more than one identified pathogen (if tick is positive for both *Borrelia* and *B. burgdorferi* or *Borrelia* and *B. miyamotoi* that is only one identified pathogen).

### Modeling

**Fig. 5.**
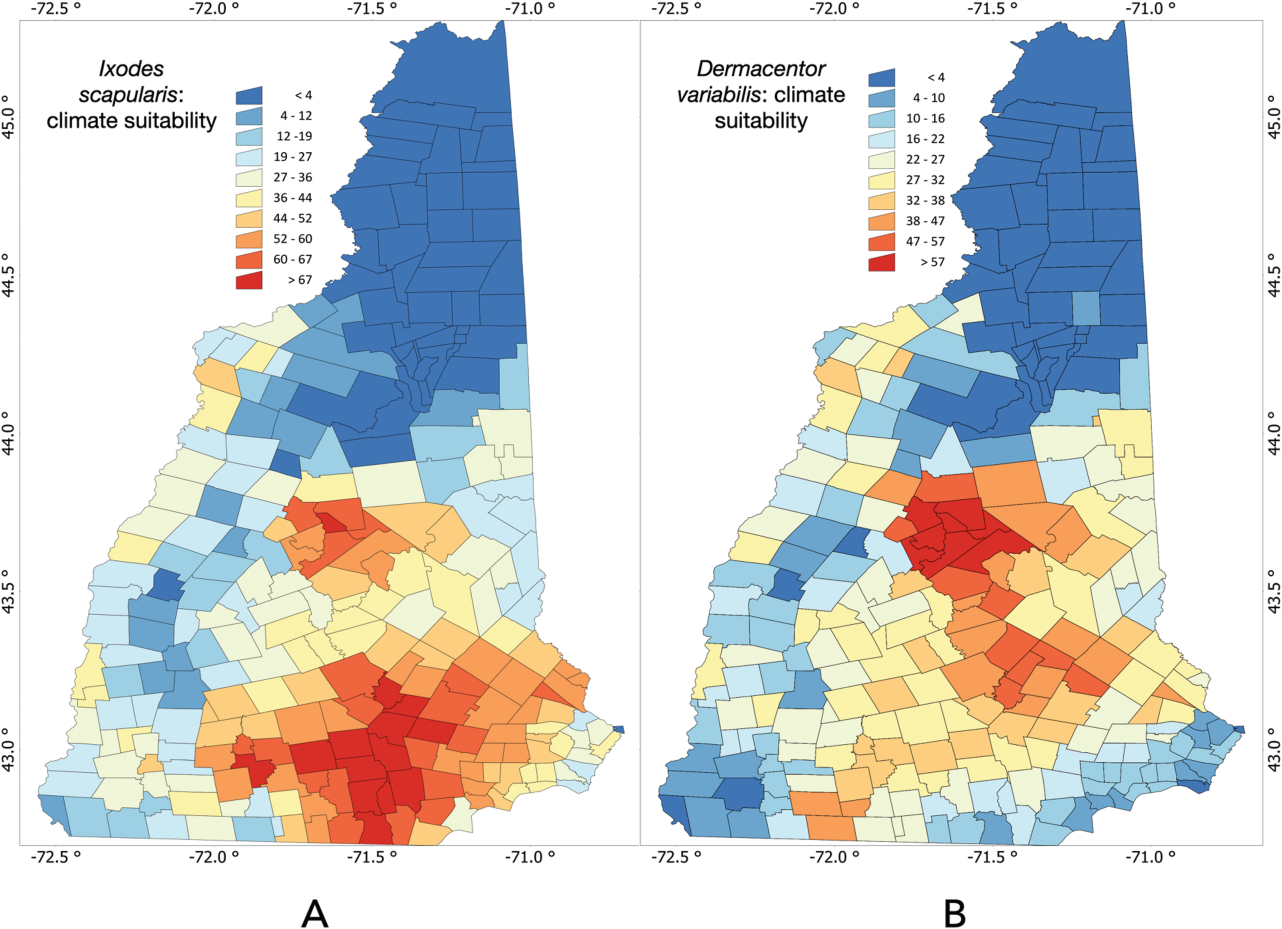
Predicted distribution of ticks within New Hampshire based on a weather-dependent model. *I. scapularis* (Fig. 5A), and D. *variabilis* (Fig. 5B), mapped as probabilities from 0 to 100 as indicated by the legends.

We modeled the distribution of both *I. scapularis* and *D. variabilis* using temperature, soil moisture, and air water vapor deficit (Fig. 5). The results obtained reflect the expected environmental suitability for both species of ticks on a scale of 0–100 based on the probability of presence. Both presence models provided a Boyce’s index greater than 0.8 (0.82 for *I. scapularis*, 0.88 for *D. variabilis*) meaning for good predictive results in the modeling protocols.

The model for *I. scapularis* predicts that the tick should be absent in northern NH, with probabilities of presence being higher in the southeastern part of the state according to a north-south gradient of increasing suitability (Fig. 5). Interestingly, there are two clear areas of high suitability, namely the southern area, and the center of the State. A completely different picture has been obtained for *D. variabilis* (Fig. 5) meaning that both species may share some portions of the habitat, but do not completely overlap in NH. *Dermacentor variabilis* is also predicted to be absent in the north of the state, but it could find a suitable weather more northern than *I. scapularis*.

## Discussion

The current study used community science to address the identification of ticks submitted by people in the state of New Hampshire (USA) following an advertised program through news and social media, resulting in a total of 14,293 ticks, in the period 2018–2021, of which 6,599 had a ‘human activity’ associated (according to returned questionnaires). The predominant ticks submitted were *I. scapularis* and *D. variabilis*, but additional species were also collected The identification of a single *I. pacificus* is also negligible and unusual as *I. pacificus* is more common to the western United States (Hahn et al. 2016, Xu et al. 2019, Porter et al. 2021), perhaps introduced from traveling, a matter not included in our questionnaire. According to Sonenshine (2018) the records of *A. americanum* in NH far exceed its northern distribution range, but the few specimens submitted preclude further conclusions about the spread of this tick into northern latitudes; these records were probably casual introductions carried by wildlife, since some species of birds may be hosts of this tick (Allan et al. 2010) or translocation from traveling participants. Yet, *A. americanum* is frequently identified in the state of Maine, as it has a more active state surveillance program than NH (Keirans et al. 1998b, Lado et al. 2020).

Prior research from passive surveillance studies is that they primarily focus on one species of tick (*I. scapularis*) and/or one pathogen (*B. burgdorferi s.l*.) (Ogden et al. 2018, Porter et al. 2019, 2021, Eisen and Paddock 2020). Yet, our research primarily focused on both *I. scapularis* and *D. variabilis* because their overwhelmingly high relative abundance in the submitted samples. When we began this project in 2017, the communication campaign and the number of ticks received was considerably fewer than for the other years. We also consider that results from the year 2020 could be affected in some way because of the ongoing SARS-CoV-2 pandemic. This is impossible to verify, but the number of ticks submitted in 2020 is slightly lower than for other years. Even if we consider these challenges, we can pinpoint tick distribution across large regions (but see Eisen and Eisen 2021, for the pros and cons of the method) and we think that the number of ticks is high enough to establish conclusions. In collecting and tracking patterns of both *I. scapularis* and *D. variabilis*, each species has unique seasonality and distribution patterns, and pathogen’s prevalence as compared to each other. Thus, the analysis of both species next to each other helps make our results novel, helping people to better prevent themselves from tick encounters.

We received significantly more *D. variabilis* than *I. scapularis*, which is supported by other studies reporting *D. variabilis* as the predominant *Dermacentor* spp. within the northern part of the United States (Dergousoff et al. 2013, Boorgula et al. 2020, Duncan et al. 2021). Both *D. variabilis* and *I. scapularis* have different periods of activity in our surveys. The adults of *D. variabilis* have a unimodal cycle, extending its activity in the target region approximately in the weeks 16–31 and showing only one major peak, most commonly around week 21. A higher number of submissions of *D. variabilis* versus *I. scapularis* happened in 2019 and 2021. We consider the reduction in *D. variabilis* submissions in 2018 and 2020 because *D. variabilis* follows a 2-yr cycle, the first year with immatures feeding on small wild animals (going unnoticed by contributors), the second with adults biting humans and other hosts. If another factor would affect the submitting efforts, the observed decrease would also be mirrored by data on *I. scapularis,* a fact not detected in our series of data. For both tick species, we found that small differences may occur between consecutive years because many factors, including (but not exclusive to) the weather of the previous autumn-winter, the spring rise of temperature, the abundance of hosts, and probably the composition of vertebrates’ communities. *Dermacentor variabilis* is also larger than *I. scapularis*, thus it is easier to find crawling or biting.

The bimodal activity of *I. scapularis* has been already reported on many occasions, although it is commonly driven by the dominant climate (Ogden et al. 2018). In our results, the total number of submissions of *I. scapularis* did not experience large changes among years. The autumn-winter peak did have a larger variability in the number of submitted specimens of *I. scapularis* than during other seasons of the year, as already demonstrated for other regions (Ostfeld et al. 1996, Ogden et al. 2006). Our short series of data did not confirm an expansion of the activity period of *I. scapularis,* but it is indicated that this species is active all year round with clear peaks of more abundance. Both species of ticks have overlapping peaks in late spring and early summer. People should take preventive measures, and routinely perform tick checks during the months of April through August due to high tick abundance.

Human activity influences the risk of an individual to be bitten by a tick. We aimed to address the most basic classification of the literally hundreds of activities reported by participants. Salkeld et al. (2019) also manifested the difficulty in relating a human activity with the tick bite; however, a different pattern emerged from the one noted for *I. scapularis* by Mead et al. (2018) or Porter et al. (2019) who pointed out a higher number of contacts in people’s yards, in contrast with the low number of exposures detected in forest-associated recreations. This large difference of results may be due to different methods of classification of the human activities, since Mead et al. (2018) only reported ‘outdoors in public spaces’, which includes several of our categories. This is a complex topic because the time elapsed between the tick bite and its finding affects what an individual records as the activity. Similar to the reports, within this project we did observe a higher tick encounter for human activities associated with walking/ hiking and backyard activities as supposed to activities in an urbanized location or around water. The smallest number of tick encounters was associated with human bicycling.

Our reported prevalence results of *Borrelia* spp., *B. burgdorferi s.l., B. miyamotoi*, *B. mayonii, A. phagocytophilum*, and *Ba. microti* harbored by *I. scapularis* are not uncommon in comparison with other published reports in the region (Schulze et al. 2013, Johnson et al. 2017, Sánchez-Vicente et al. 2019) or for the complete country (Porter et al. 2021). The detection of up to 37% of ticks positive to the DNA of *B. burgdorferi s.l.* is far higher than the relatively low values found for *Ba. microti* or *A. phagocytophilum*, although the rise of *Ba. microti* has been reported (Diuk-Wasser et al. 2014). Similar percentages of *I. scapularis* containing *B. burgdorferi s.l.* have also been previously observed (Little et al. 2019, Xu et al. 2019). *Borrelia miyamotoi* was present in 1% of the tested ticks. The co-infection of ticks with more than one pathogen was 7%, in line with other reports (Benach et al. 1985, Sánchez-Vicente et al. 2019, Milholland et al 2021). These findings support the idea of NH as an area of high risk for *Borrelia* spp., a fact to be considered when planning and implementing informative alerts to the public. *Dermacentor variabilis* was tested for *F. tularensis* and *R. rickettsii*, pathogens commonly found in this tick, but analyses yielded only 1 out of 1,041 tested *D. variabilis*.

An interesting point noticed in this study is the lack of submissions of both *I. scapularis* and *D. variabilis* from the northern parts of the state. Reasons for the lack of submissions from northern regions may include (a) the poor impact of our project advertisements in the northern region, (b) a lower population in the northern parts of the state decreasing the probabilities of participation, (c) the lack of important roads in the region, preventing humans to move easily among different areas of this part of the state, then impeding the contact with ticks, or (d) the actual absence of these ticks in the area. Additionally, with our passive surveillance protocol we would expect to receive more specimens from areas with higher awareness. Yet, since we demonstrated that the distribution of ticks received is random, there is no correlation with the total population of the State, and the climate-predicted distribution of both species is different, it reduces the limitations of this protocol.

We created maps predicting the tick distributions in NH based on submissions, that were primarily associated with temperature, soil moisture, and atmospheric water vapor deficit. We did not include data on vegetation as an explanatory variable (Winter et al. 2021), because of the scale of the mapping (meters). Although produced at a different resolution, and using different explanatory variables, our map of the probability of suitable habitat for *I. scapularis* in NH fits well with the results obtained by Diuk-Wasser et al. (2010). Similar studies have also shown the effect of climate change on Lyme disease within the USA (Moore et al. 2014, Monaghan et al. 2015, Couper et al. 2021) or northeastern parts of the country (Little et al. 2019, Elias et al. 2021). The mapping efforts suggest that modeling habitat suitability for tick vectors may contribute better to epidemiological models of transmission of tick-borne pathogens. We evaluated if more ticks were received from more populated sites, a situation that could distort the purely environmental modeling; we however rejected that hypothesis as there was no correlation at the scale of the State. This precludes any *substantial* biasing effect. Then, predictive modeling using only environmental variables could be able to effectively separate the weather factors affecting tick distribution and removing the biasing effects of the human side.

The combination of active surveys, community science, and predictive mapping may be a compelling source of information, increasing the knowledge about the distribution of *I. scapularis* and *D. variabilis*; thus, improving tick bite and tick-borne disease prevention campaigns. We demonstrated that passive surveillance of ticks may be an excellent tool *if paired* with active surveys; otherwise, some ‘background noise’ (linked to sites of higher awareness) may be present in the data obtained. This passive surveillance data is extremely important, considering the scale (a complete state of USA), the fraction of costs, and the continuously updated set of data as submitted by the participants. Even with the constraints mentioned in this study, data obtained by volunteer participation may be an excellent source for tick-borne pathogens detection, even if only pinpointing the relative importance of each organism in the complete target territory.
